# Characterization of Transverse Aortic Constriction in Mice Based on the Specific Recruitment of Leukocytes to the Hypertrophic Myocardium and the Aorta Ascendens

**DOI:** 10.1155/2021/1376859

**Published:** 2021-11-03

**Authors:** Jan Lukas Kleiner, Odilia Köpke, Anton Faron, Yunyang Zhang, Jan Cornelssen, Mark Coburn, Stilla Frede, Lars Eichhorn, Christina Katharina Weisheit

**Affiliations:** ^1^Department of Anesthesiology and Intensive Care Medicine, University Hospital Bonn, Germany; ^2^Department of Obstetrics and Gynecology, Marienhospital, Bonn, Germany; ^3^Department of Diagnostic and Interventional Radiology, University Hospital Bonn, Germany

## Abstract

Transverse aortic constriction (TAC) is a model that mimics pressure overload-induced left ventricular (LV) hypertrophy in mice. Alterations in immune cell functionality can promote cardiac and vascular remodeling. In the present study, we characterized the time course in innate immune cell dynamics in response to TAC in the different tissues of mice. It was determined whether TAC induces a characteristic leukocyte-driven immune response in the myocardium, aorta ascendens and descendens, spleen, blood, and draining lymph nodes supported by cytokine-driven chemotaxis in mice at 3, 6, and 21 days following surgery. We used complex flow cytometry staining combinations to characterize the various innate immune cell subsets and a multiplex array to determine cytokine concentrations in the serum. The results of the current study indicated that leukocytes accumulate in the myocardium and aorta ascendens in response to TAC. The leukocyte dynamics in the myocardium were dominated by the Ly6C^low^ macrophages with an early accumulation, whereas the response in the aorta ascendens was characterized by a long-lasting proinflammatory phenotype driven by Ly6C^high^ macrophages, neutrophils, and activated DCs. In contrast to the high-pressure environment of the aorta ascendens, the tissue of the aorta descendens did not react to TAC with any leukocyte increase. The levels of proinflammatory cytokines in the blood were elevated in response to TAC, indicating a systemic reaction. Moreover, our findings strongly suggest that cardiac macrophages could origin from splenic pools and reach the site of the inflammation via the blood. Based on the current findings, it can be concluded that the high-pressure conditions in the aorta ascendens cause a characteristic immune response, dominated by the accumulation of leukocytes and the activation of DCs that varies in comparison to the immune cell dynamics in the myocardium and the aorta descendens.

## 1. Introduction

Chronic pressure overload induces left ventricular (LV) hypertrophy and consequently results in congestive heart failure. This process is driven by the immune response and is known to influence the outcome and quality of life. In sterile inflammations, the innate immune system is activated by danger-associated molecular patterns (DAMPs) that are released by injured or necrotic cells and damaged extracellular matrix. DAMPs can activate the response of leukocytes via toll-like receptor signaling [1]. Our recent studies indicated that LV hypertrophy induced by pressure overload provokes an immune response which is dominated by anti-inflammatory macrophages [2]. These cardiac Ly6C^low^ CX3CR1^+^ macrophages contribute to myocardial hypertrophy and impaired heart function following pressure overload in a mouse model of transverse aortic constriction (TAC) [3].

As previously shown, our TAC model causes LV hypertrophy and heart failure in mice [3]. Moreover, we assume that the narrowing of the aortic lumen provokes a high-pressure (in front of the constriction) and a low-pressure part (behind the constriction) of the aorta that are characterized by a specific leukocyte response. The TAC model can be used to analyze the high-pressure and low-pressure parts of the aortic tissue in the same animal. The aim of the present is to further investigate the local and systemic inflammatory response induced by TAC. Besides, the present study investigates not only the immune response in the myocardium but also the ongoing inflammatory processes in the aorta, spleen, lymph nodes, and blood.

The most important cells of the innate immune system involved in cardiac remodeling processes are monocytes/macrophages, neutrophils, and dendritic cells (DCs) [4]. Murine monocytes and macrophages can be classified by either the Ly6C^low^ or Ly6C^high^ surface expression in the patrolling monocytes or anti-inflammatory macrophages (Ly6C^low^ in mice and CD14^low^CD16^+^ in humans), which survey the vascular lumen, clear cellular debris, and contribute to remodeling processes, as well as in the inflammatory monocytes and macrophages (Ly6C^high^ in mice and CD14^high^CD16^−^ in humans), which secrete a variety of proinflammatory cytokines and maintain inflammation [5, 6]. Our previous work had shown that these two distinct macrophage subsets cross-talked and orchestrated the immune defense against bacterial urinary tract infection, by regulating neutrophil migration into the infected uroepithelium [7]. In the context of cardiovascular diseases, we elucidated the influence of pattern recognition receptors expressed by cardiac macrophages and circulating leukocytes to cardiac remodeling following myocardial infarction and pressure overload induced LV hypertrophy, using our TAC model [2, 3, 8, 9].

We recently published that cardiac macrophages do not replenish by local proliferation in response to pressure overload-induced LV hypertrophy but appear to be recruited via the chemotaxis of CCL2 to the site of the myocardial inflammation [3]. In this context, the source of the recruited monocytes has not been further investigated.

Under steady-state conditions, the resident cardiac DCs represent approximately 1% of the total cardiac leukocytes and can be divided into CD103^+^ and CD11b^+^ cells in a model of viral myocarditis [10]. In the context of MI, the DCs infiltrated the infarcted heart, internalized locally released cardiomyocyte-derived antigens, and migrated into the mediastinal lymph nodes, where they presented antigen-derived peptides and stimulated T cells [11].

In the present study, we focused on the dynamics of leukocyte accumulation in the myocardium as well as in the ascending and descending parts of the aorta, spleen, lymph nodes, and blood to characterize the potential remote immunologic effects of TAC-induced cardiac hypertrophy.

## 2. Materials and Methods

### 2.1. Mice

Female C57BL/6J mice aged 10-14 weeks were purchased from Charles River (Wilmington, MA, USA). The mice were kept under specific pathogen-free conditions in isolated, ventilated cages with free access to water and food. All animal experiments were approved by the governmental ethics board at the Ministry of Nature, Environment and Consumer Protection of the German state of North Rhine Westphalia (LANUV Recklinghausen permit numbers 84-02.04.2011.A313, 84-02.04.2016.A374) and were supervised by the central animal facilities of the medical faculty of Bonn (HET, Venusberg-Campus 1, Bonn, Germany). All surgical interventions were performed under anesthesia and analgesia as described below, and all efforts were made to minimize the suffering of the mice.

### 2.2. Transverse Aortic Constriction (TAC)

TAC was performed as published previously [2]. In short, mice were anesthetized with isoflurane (2 vol%) and intubated with an intubation cannula (OD 1.2 mm). The respiratory rate was set to 150/min and the tidal volumes to 8-10 ml/kg body weight using a small animal ventilator (Harvard Apparatus; Holliston, MA, USA). A 27 G spacer was used to standardize the degree of aortic constriction. The sham control animals underwent intubation and surgery except that the suture around the aorta was not tight. For postoperative pain management, buprenorphine was administered (0.1 *μ*g/g subcutaneously) every 8 h for the next 3 days after surgery. The mice were sacrificed 3, 6, and 21 days following surgical intervention.

### 2.3. Flow Cytometry

The immune cells were isolated from the blood, spleen, lymph node, aorta, and LV tissue. Peripheral blood (100 *μ*l) was collected in tubes with ethylenediaminetetraacetic acid (EDTA) (Sigma Aldrich, St. Louis, MO, USA). The spleen tissue and lymph nodes were mechanically homogenized and filtered via 80 *μ*m meshes. The spleen samples and blood were treated with RBC lysis buffer (Thermo Fisher Scientific, Waltham, MA, USA) to eliminate erythrocytes. Whole hearts were flushed with PBS, and the heart tissue and parts of the aorta were minced into small pieces using a single-edged blade and digested in gently agitated RPMI medium with 1 mg/ml collagenase 2 (Sigma Aldrich) and 1 mg/ml DNAse I (Sigma Aldrich) at 37°C for 60 min.

Unspecific Fc-receptor binding was blocked incubating the cells with anti-mouse CD16/32 (BD Biosciences, Franklin Lakes, NJ, USA) for 10 min at 4°C. The cells were stained with anti-mouse fluorochrome-conjugated antibodies in panel-appropriate combinations in the dark for 20 min at 4°C with following antibody clones from Thermo Fisher (Waltham, MA, USA), (i.e., CD45 (30-F11), CD11b (ITGAM), CD115 (c-fms), F4/80 (BM-8), Ly6C (HK1.4), Ly6G (1A8)) and BioLegend (San Diego, CA, USA) (i.e., CD103 (2E7), CD11c (Cr4)). We performed live/dead staining using Hoechst 33258 in a dilution of 1 *μ*l/100 ml (Thermo Fisher). Flow cytometry was performed on a BD Canto and a BD LSR Fortessa, and the data were analyzed using the Flow-Jo software (BD Bioscience).

### 2.4. Cytokine Measurement

The cytokine concentrations in the serum of the mice were measured using a custom-made FlowCytomix Multiple Analyte Detection System (Thermo Fisher, formerly eBioscience). This bead-based immunoassay for the simultaneous detection of the cytokines in one sample was performed according to the manufacturer's instructions. As suggested by the manufacturer, 25 *μ*l of mouse serum was used. The data were evaluated using the FlowCytomix Pro Software.

### 2.5. Statistical Analysis

Appropriate assumptions of the data (e.g., normal distribution or similar variation between experimental groups) were examined before the statistical tests were conducted. Student's *t*-tests were performed whenever two groups were compared, whereas one-way or two-way analyses of variance followed by Tukey's test were performed for multiple comparisons. The analysis was performed using Prism 8 (GraphPad Software, Inc., La Jolla, CA, USA). The results were provided as mean ± standard deviation (SD) unless noted otherwise; a *P* value of < 0.05 was considered statistically significant.

## 3. Results

### 3.1. The Effect of TAC on the Immune Responses in the Myocardium and the High-Pressure and Low-Pressure Parts of the Aorta

Our recent studies highlighted that LV hypertrophy caused by pressure overload induces a complex immune response in the myocardium [2]. Moreover, we focused on the immune response in the LV tissue and did not investigate the systemic or remote immune response. In the present study, we examined the migration pattern of immune cells to the ascending and descending parts of the aorta, draining lymph nodes, blood, spleen, and LV tissue in response to TAC, which has not been determined systematically in previous studies.

**Figure 1:**
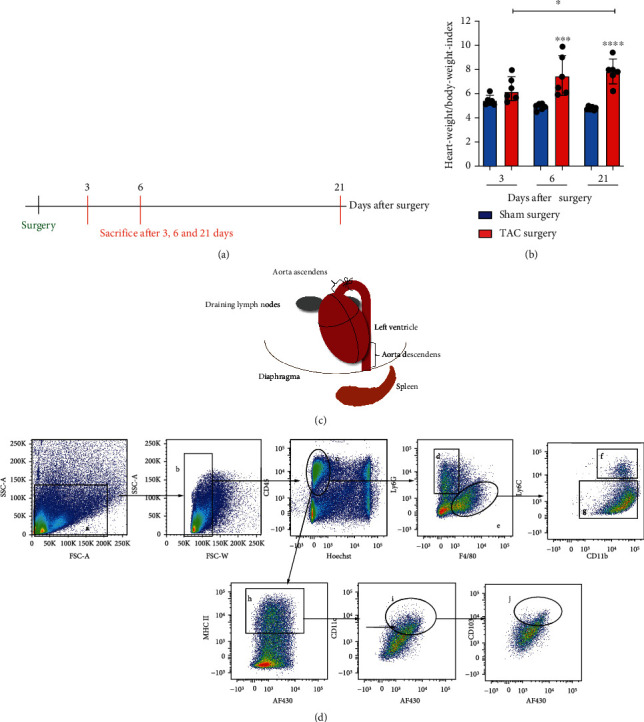
Methodological procedure and definition of the immune cell subsets. (a) We sacrificed the mice 3, 6, and 21 days after TAC or sham surgery. (b) Heart-weight/body-weight-index in mg/g was calculated 3, 6, and 21 days after TAC or sham surgery. (c) In addition to the LV tissue, we analyzed the tissue of the aorta ascendens, aorta descendens, spleen, blood, and heart draining lymph nodes. (d) The gating strategy for flow cytometry analyses was performed as illustrated for the tissue of the aorta ascendens. All cell-like events (a) were isolated by using the forward scatter area versus sideward scatter area. Doublets were excluded using forward scatter area versus sideward scatter width (b). We defined the living immune cells (c), using CD45 staining versus Hoechst staining. The CD45^+^ immune cells were further classified by staining for the surface markers Ly6G and F4/80 to determine neutrophils (d) and macrophages (e). Next, we checked for the Ly6C surface expression of the macrophage population as a function of CD11b to differentiate between the Ly6C^high^ (f) and the Ly6C^low^ macrophages (g). We analyzed the DC compartment based on the population of the CD45^+^ cells (c) and stained for MHC-II to identify MHC-II^+^ immune cells (h). We used CD11c as a function of the autofluorescence channel AF430 for the definition of classical dendritic cells (i). The cDCs were further discriminated according to the CD103 surface expression (j).

In this project, we performed TAC or sham surgery and analyzed the immune response in the course of the disease at 3, 6, and 21 days after intervention (Figure 1(a)). Our model of TAC induced LV hypertrophy was evaluated by calculating the heart-weight/body-weight-index of the mice. Hypertrophy of the hearts becomes significant at days 6 and 21 after TAC compared to the controls (Figure 1(b)). In addition to the myocardium, we investigated the immune cell dynamics in the ascending and descending parts of the aorta, draining lymph nodes, spleen, and blood, which allowed us to describe the migration and recruitment processes provoked by TAC (Figure 1(c)). The immune cell subsets and activation markers were determined via flow cytometry. Our gating strategy was based on the guidelines for the use of flow cytometry in immunology [12] and was optimized and refined with the help of our flow cytometry core facility (Figure 1(d) and Supplemental Figure 1).

**Figure 2:**
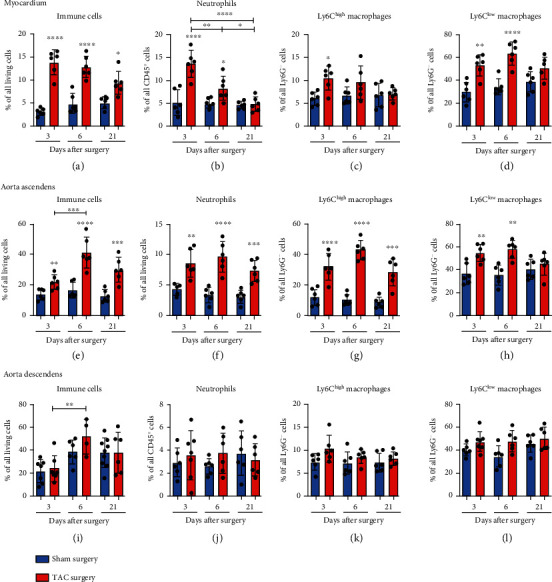
Innate immune response in the myocardium and the ascending and descending parts of the aorta. (a)–(l) The CD45^+^ immune cells, neutrophils, Ly6C^low^, and Ly6C^high^ macrophages in the tissue of the LV, the aorta ascendens and descendens of the mice were quantified via flow cytometry. The particular cell subsets were defined as follows: neutrophils as CD45^+^ F4/80^−^ Ly6G^+^ and macrophages as CD45^+^ F4/80^+^ Ly6G^−^. Moreover, the Ly6C^high^ and Ly6C^low^ macrophages were further discriminated according to the respective Ly6C surface expression. ^∗^The above individual columns indicate the significant differences between the TAC and respective sham group; ^∗^*P* < 0.05, ^∗∗^*P* < 0.01, ^∗∗∗^*P* < 0.001, and ^∗∗∗∗^*P* < 0.0001.

The characterization of the immune cell composition in the myocardium indicated a significant increase in the proportion of immune cells at all three time points (Figure 2(a)). The percentage of neutrophils and Ly6C^low^ macrophages was significantly elevated at days 3 and 6 after TAC compared with the controls (Figures 2(b) and 2(d)). The Ly6C^high^ macrophages significantly accumulated only at day 3 after TAC compared to controls (Figure 2(c)). Next, it was determined whether and how our model of TAC-induced LV hypertrophy affected the high-pressure and low-pressure parts of the aorta to determine specific immunological signatures.

Flow cytometry analyses performed at days 3, 6, and 21 after TAC or sham surgery revealed that TAC surgery directly influenced the immune reaction in the ascending aortic tissue compared with that in the descending part of the aorta. The immune response in the ascending part of the aorta was characterized by a significant increase in CD45^+^ immune cells at days 3, 6, and 21 after TAC compared to controls. The increase in CD45^+^ immune cells was significantly higher at day 6 compared to day 3 indicating that the inflammation increases in the first 6 days (Figure 2(e)). Especially neutrophils and Ly6C^high^ macrophages invaded the aortic tissue in the high-pressure environment, reaching the level of significance at all three time points in comparison with the sham animals (Figures 2(f) and 2(g)). The Ly6C^low^ macrophages significantly increased in response to TAC at days 3 and 6 compared with controls decreasing to sham level at day 21 (Figure 2(h)). In the LV tissue, the neutrophils and Ly6C^low^ macrophages dominated the immune response with significantly elevated cell proportions at days 3 and 6 after TAC compared with the control groups, a situation which was mirrored in the ascending part of the aorta. The Ly6C^high^ macrophage levels were significantly increased at day 3 only in the myocardium, whereas the reaction in the aorta ascendens lasted for 21 days (Figures 2(c) and 2(g)). In the descending part of the aorta, a significant increase in immune cells was observed only at day 6 after TAC compared with day 3 which we could not directly break down to any of the analyzed innate immune cell subsets (Figure 2(i)). The proportion of the different leukocyte subsets did not vary in response to TAC (Figures 2(j)–2(l)). The descending part of the aorta did not reflect the immune response of the myocardium but reacted to the surgical intervention and maybe changes in the blood flow f.e. turbulences that may result in an increase in immune cells in general.

### 3.2. TAC Provokes an Immune Response in Spleen and Blood

Certainly, monocytes and macrophages play important roles in the orchestration and maintenance of the cardiac inflammation in the development of pressure overload-induced LV hypertrophy. Our recent findings demonstrated that CCL2-driven chemotaxis plays an important role in the recruitment of cardiac macrophages to the site of cardiac injury [3]. The remote activation of the splenic immune response provoked by TAC has not been systematically studied yet. In post-MI inflammation and remodeling, it is already known that cardiac macrophages originate mostly from splenic pools and are transported via the blood to the site of the inflammation [13]. However, the immune response in MI and LV hypertrophy with a biphasic response after MI differs from the chronic response with dominating Ly6C^low^ macrophages in the myocardium after TAC [2]. Considering this, we determined whether the immune cell composition of the spleen and blood is affected from TAC, and whether the splenic myeloid cell pool might serve as a source to supply the cardiac immune response in our TAC model.

**Figure 3:**
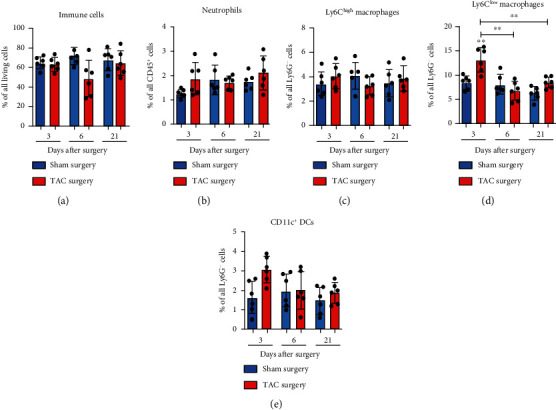
Innate immune response in the spleen. (a)–(e) The CD45^+^ immune cells, neutrophils, and Ly6C^low^ and Ly6C^high^ macrophages, and DCs in the spleen of the mice were quantified via flow cytometry. The particular cell subsets were defined as follows: neutrophils as CD45^+^ F4/80^−^ Ly6G^+^ and macrophages as CD45^+^ F4/80^+^ Ly6G^−^. Moreover, the Ly6C^high^ and Ly6C^low^ macrophages were further discriminated according to the respective Ly6C surface expression, and the DCs were defined as CD45^+^ F4/80^−^ Ly6G^−^and CD11c^+^. ^∗^The above individual columns indicate the significant differences between the TAC and respective sham group; ^∗^*P* < 0.05, and ^∗∗^*P* < 0.01.

The results of the present study showed a decrease in splenic immune cells at day 6 after TAC compared with the sham group, not reaching the level of significance (Figure 3(a)). The kinetics of the Ly6C^low^ macrophages in the spleen showed a significant increase at day 3 compared with the sham controls and both other time points that were paralleled by an increase in DCs (Figures 3(d) and 3(e)). The first increase of splenic Ly6C^low^ macrophages was followed by a decline to sham level at days 6 and 21 after TAC (Figure 3(d)). The quantification of splenic Ly6C^high^ macrophages and neutrophils did not reveal any changes following TAC (Figures 3(b) and 3(c)). These findings showed that there is a remote reaction of the immune system in the spleen triggered by TAC.

**Figure 4:**
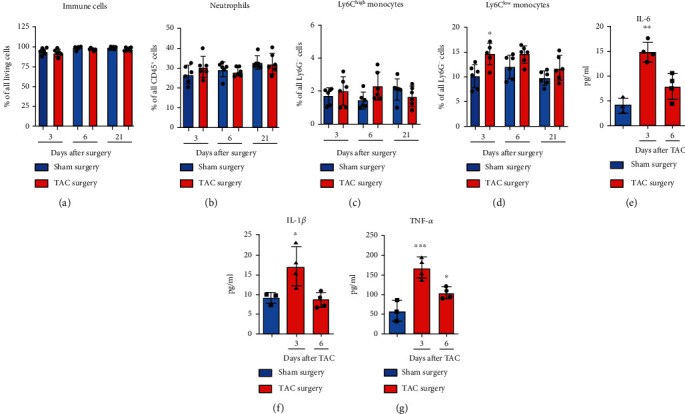
Innate immune cell quantification and cytokine levels in response to TAC in the blood. (a)–(d) The CD45^+^ immune cells, neutrophils, and Ly6C^low^ and Ly6C^high^ monocytes in the blood of the mice were quantified using flow cytometry. The particular cell subsets were defined as follows: neutrophils as CD45^+^ CD115^−^ Ly6G^+^ and monocytes as CD45^+^ CD115^+^ Ly6G^−^. In addition, the Ly6C^high^ and Ly6C^low^ monocytes were further discriminated according to the respective Ly6C surface expression. (e)–(g) The cytokine levels of IL-6, IL-1*β*, and TNF-*α* in the serum of the mice were measured at days 3 and 6 after TAC or after day 3 after sham surgery. ^∗^The above individual columns indicate the significant differences between the TAC and respective sham group; ^∗^*P* < 0.05, ^∗∗^*P* < 0.01, and ^∗∗∗^*P* < 0.001.

From the spleen, the immune cells are transported via the circulation to the site of inflammation and injury. We suggested that TAC provoked changes in the amount of immune cells in the blood caused by the release of monocytes from the spleen for the recruitment of monocytes to the injured heart. Our flow cytometry-based quantification approach revealed a significant increase only in the population of the Ly6C^low^ monocytes at day 3 after TAC compared with the sham group (Figure 4(d)). No significant increase was detectable at days 6 and 21 after TAC. The reaction of neutrophils did not alter in response to TAC, and the observed changes in the immune cell subsets did not cause alterations of the total immune cell compartment (Figure 4(a)). In addition to the flow cytometry studies, we determined the levels of proinflammatory cytokines in the serum of TAC and sham mice as indicators of systemic inflammation. The results of the current study indicated a significant increase in IL-6 and IL-1b at day 3 after TAC compared with the control group (Figures 4(e) and 4(f)). The concentration of TNF-a was significantly elevated at days 3 and 6 in response to TAC compared with the sham group (Figure 4(g)). This data demonstrated a systemic immunological reaction reflected in the blood by an increase in proinflammatory cytokines.

The analysis of draining lymph nodes with regard to changes in the leukocyte composition did not exhibit noteworthy changes in response to TAC (data not shown).

### 3.3. Dendritic Cells Are Activated in Response to TAC in the Myocardium and Aorta Ascendens

Recently, a published work demonstrated that TAC-induced LV hypertrophy is associated with the accumulation of CD11c^+^ MHC-II^+^ cells in the LV, spleen, and blood [14]. Based on these findings, we decided to take a closer look at both the DC activation pattern and subtypes in the myocardium, aorta ascendens and descendens, and draining lymph nodes.

**Figure 5:**
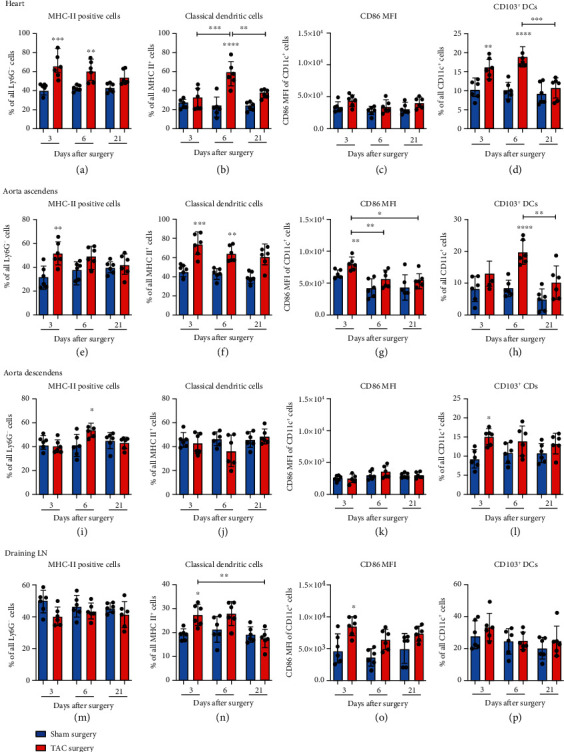
TAC provoked the response of the DC compartment in the myocardium, aorta ascendens, aorta descendens, and draining lymph nodes. (a)–(p) The total of the MHC-II^+^ cells was determined from the fraction of CD45^+^ Ly6G^−^ cells. The population of cDCs was defined using the CD11c surface staining. CD86 MFI was calculated from the population of all cDCs. The population of CD103^+^ cDCs was determined based on the results of the CD103 surface staining. ^∗^The above individual columns indicate the significant differences between the TAC and respective sham group; ^∗^*P* < 0.05, ^∗∗^*P* < 0.01, ^∗∗∗^*P* < 0.001, and ^∗∗∗∗^*P* < 0.0001.

Our analyses revealed that the percentage of MHC-II^+^ cells was significantly elevated in the myocardium 3 and 6 days after TAC compared with the respective control group (Figure 5(a)). These MHC-II^+^ cells were further subdivided into classical DCs according to the CD11c surface expression. The amount of classical DCs (cDCs) significantly increased at day 6 compared with that at days 3 and 21 after TAC and the respective controls (Figure 5(b)). However, the activation of these cells in the myocardium, measured by CD86 MFI, did not show any changes in response to TAC (Figure 5(c)). Since the majority of the aortic CD11c^+^ MHC-II^+^ DCs showed no staining for E-cadherin ligand CD103, a small CD103^+^ DC population could be confined in the myocardium (Figure 5(d)). In our model, the CD103^+^ DCs increased at days 3 and 6 after TAC compared with the control groups. At day 21, the increase in CD103^+^ DCs returned to the initial level (Figure 5(d)).

In addition to the myocardium, we examined the DC response in the ascending, high-pressure part of the aorta. In response to TAC, we observed a significant increase in MHC-II^+^ cells at day 3 after TAC compared with the sham group (Figure 5(e)). The proportion of cDCs was significantly elevated at days 3 and 6 after TAC compared with the respective sham animals and presented with an earlier onset compared with our observations in the myocardium (Figure 5(f)). We determined a significant increase in the activation marker CD86 at day 3 after TAC compared with the sham group and with the TAC groups at days 6 and 21 in the aorta ascendens (Figure 5(g)). The CD86 MFI indicated a higher activity level of cDCs in the aorta ascendens after TAC (mean: 5501-8004) compared with that in the myocardium (mean: 3447-4414) (Figures 5(k), 5(g), and 5(o) and Supplementary Figure 2). The amount of CD103^+^ DCs was significantly increased in response to TAC at day 6 compared with the control group and the 21-day TAC group in the aorta ascendens (Figure 5(h)).

In the tissue of the aorta descendens, less changes were observed in the population of DCs. The findings of the present study showed a significant increase in MHC-II^+^ cells at day 6 after TAC compared with the sham group (Figure 5(i)). The kinetics of cDCs and DC86 MFI did not reach the level of significance at any time point (Figures 5(j) and 5(k)). The mean of the CD86 expression pattern did not exceed the mean of 3513 at day 6 after TAC, which represents a low activation level compared to the CD86 MFI measured on the surface of the cDCs in the aorta ascendens and the draining lymph nodes (Figures 5(c) and 5(g) and Supplementary Figure 2). However, we determined a significant increase in CD103^+^ DCs at day 3 after TAC compared with the sham group (Figure 5(l)).

The examination of the draining lymph nodes with focus on the DC dynamics in response to TAC revealed a significant increase in cDCs at day 3 after TAC compared with the respective control group and the 21-day TAC group (Figure 5(n)). CD86 MFI was significantly elevated at day 3 after TAC in comparison with the respective control group and presented with a higher activation level (mean: 6428 at day 6 to 8519 at day 3) compared with the myocardium and the aorta descendens (Figure 5(o)).

This finding indicated an influence of TAC on the population of DCs and the activation status as well as on the DC subsets in the myocardium, aorta ascendens and descendens, and draining lymph nodes.

## 4. Discussion

In the present project, we further investigated the immune response in the myocardium, aortic tissue, spleen, blood, and lymph nodes following TAC in mice. TAC is a commonly and frequently used animal model for the examination of pressure overload-induced LV hypertrophy. Relevant systemic immunological reactions following TAC induction are of great interest in the field of cardiovascular research [15]. Alterations in the immune cell activity and quantity can promote cardiac remodeling and functional outcome [16]. Herein, we characterized the time course in innate immune cell dynamics in response to TAC in the different tissues of mice. We investigated whether TAC induces a characteristic leukocyte-driven immune response in the myocardium, aorta ascendens and descendens, spleen, blood, and draining lymph nodes supported by cytokine-driven chemotaxis in mice in the course of the disease.

The results of the current study indicated that the dynamics of immune cells and neutrophils and Ly6C^high^ and Ly6C^low^ macrophage accumulation in the myocardium were mirrored in the aorta ascendens which is directly affected by the increase in blood pressure after TAC. We observed significantly increased amount of leukocytes at all three time points following TAC in the myocardium and the tissue of the aorta ascendens, highlighting a chronic inflammation in a duration of 21 days. The TAC model allowed the simultaneous analysis of the aortic tissue in a high- and low-pressure environment in front of and behind the knot of the suture.

We assume that the early accumulation of macrophages at day 3 in the spleen reflects the systemic inflammation in response to TAC and the decline in macrophages compared to day 3 after TAC could mirror the release of these cells to the circulation. Unfortunately, our data did not indicate a significant increase in monocytes in the blood at day 6 after TAC. This might be because monocytes were recruited from the circulation maybe in the same amount as they are released from the spleen. This observation underlined the hypothesis that in LV hypertrophy the invading monocytes were released from the spleen and transported via the blood to the site of inflammation similar to the replenishment of monocytes following MI [13]. Our recently published findings demonstrated that the peak of inflammatory activity is at day 3 and is paralleled by the high levels of CCL2 in the myocardium, a chemokine, which is known to attract CCR2^+^ monocytes from the circulation [3]. Besides, we could already show by means of BrdU analyses that there is no local proliferation of macrophages in the myocardium in response to TAC [3]. This supports the idea that macrophages are recruited to the heart in the development of LV hypertrophy. Moreover, the present study investigated the systemic immune response expressed by the increased levels of proinflammatory cytokines in the serum of the mice. The findings that we already published were focused on elucidating the cytokine expression pattern in the myocardium following TAC and confirmed the newly acquired data [3]. We suggest that tissue-resident endothelial and immune cells account for the increase in proinflammatory cytokines and induce a systemic immune response to attract immune cells in response to TAC.

The analysis of the DC dynamics in the myocardium, aortic tissue, blood, lymph nodes, and spleen in response to TAC allows to determine the role of DCs in the modulation of sterile inflammations. Moreover, we identified the DCs based on the surface staining of F4/80, CD11c, MHC-II, and CD103. In the myocardium, the MHC-II^+^ CD11c^+^ DCs have accumulated following TAC and peaked at day 6, whereas in the aorta ascendens, the proportion of DCs significantly increased already at day 3, and the aortic DCs presented with a significantly higher activation level based on CD86 MFI in comparison with the DCs isolated in the myocardium and aorta descendens. This finding indicates that inflammation and the high-pressure environment in the ascending part of the aorta in our TAC model induced a DC response that was more pronounced than that in the myocardium. The chronic remote inflammation induced in the heart provoked a later onset of the DC response with less activated DCs. We assume that the increase in DCs in the aorta ascendens, which is highly affected by the onset of high-pressure after TAC, contributed to the inflammation process maybe through the breakdown of vascular tolerance as in Kawasaki disease [17]. The finding confirming that CD86 expression was much higher in the DCs found in the aorta ascendens than in the myocardium or the aorta descendens supported the hypothesis that DCs are highly involved in vascular inflammation in response to TAC. The higher activation level of CD11c^+^ cDCs in control animals suggests a reaction to the sham intervention f.e. at day 3 in the sham group but might also indicate that CD86 expression is elevated under steady-state conditions in the lymph nodes. Additionally, in the group of the MHC II^+^ CD11c^+^ DCs, we observed 15% to 20% of CD103^+^ DCs after TAC in the myocardium and the aorta ascendens. CD103, a ligand for E-cadherin expressed by most epithelial cells and also a marker for CD11b^−^ DCs in many tissues, was shown to originate from the aortic sinus [18], a region which was directly affected by the TAC surgery in our model. In the draining lymph nodes, we observed a relevant increase in the population of cDCs and at day 3 an early elevation in cell activation. We hypothesize that the DC response in the draining lymph nodes played a critical role in the orchestration of the immune response following TAC in the aorta and the myocardium and contributed to T cell activation. T cells are known to promote nonischemic heart failure, and the specific antigen recognition of CD4 T cells appears to be crucial for the progression from compensated cardiac hypertrophy to heart failure [19].

Our analyses of the aorta descendens did not reveal any significant changes in the immune response following TAC which might be due to the fact that the tissue was not directly and even remotely affected.

The TAC model used in this project mirrors the situation of patients suffering from diseases such as chronic arterial hypertension or aortic valve stenosis that result in LV hypertrophy. It was considered that the acute onset of pressure overload in the TAC model did not perfectly reflect the patients' clinical course in which the pressure overload slowly evolves over the years leading to LV hypertrophy and heart failure. Despite the limitations of the current study, the model of TAC in mice has been extensively used to examine signaling pathways that contribute to adverse cardiac remodeling and hypertrophy in the context of pressure overload [15]. The observations collected in this study promote a model that can be used to compare high-pressure and low-pressure conditions in the aortic tissue in parallel in the same animal.

## 5. Conclusion

Based on the findings of the current study, it is likely that the monocytes that have accumulated in response to TAC in the myocardium originate from splenic pools and reach the site of the inflammation via the blood driven by chemotaxis. The high-pressure conditions in the aorta ascendens cause a characteristic immune response, dominated by the accumulation of leukocytes that varies depending on the immune cell dynamics in the myocardium and the aorta descendens.

## Data Availability

The authors confirm that the data supporting the findings of this study are available within the article [and/or] its supplementary material.

## Abbreviations

DAMPs: Danger-associated molecular patterns
DC: Dendritic cell
FACS: Fluorescence-activated cell sorting
IL: Interleukin
LV: Left ventricle
MFI: Mean Fluorescence Intensitiy
MHC: Major histocompatibility complex
MI: Myocardial infarction
TAC: Transverse aortic constriction
Wt: Wild-type mice (C57BL/6).

## Supplementary Materials

Supplementary Materials Supplemental Figure 1: fluorescence minus one (FMO) controls of CD11c and CD103 staining in addition to Figure 1. Supplemental Figure 2: direct comparison of CD86 MFI of CD11c^+^ cells in heart, aorta ascendens, aorta descendens, and lymph node in addition to Figure 5.
