# Supplementary material for: Characterization of Transverse Aortic Constriction in Mice Based on the Specific Recruitment of Leukocytes to the Hypertrophic Myocardium and the Aorta Ascendens
Source: Mediators Inflamm. 2021 Nov 3;2021:1376859. doi: 10.1155/2021/1376859 (PMC8580661; doi:10.1155/2021/1376859)
Supplement: Supplementary Materials — Supplemental Figure 1: fluorescence minus one (FMO) controls of CD11c and CD103 staining in addition to Figure 1. Supplemental Figure 2: direct comparison of CD86 MFI of CD11c+ cells in heart, aorta ascendens, aorta descendens, and lymph node in addition to Figure 5. [file 1376859.f1.docx]

Supplemental material


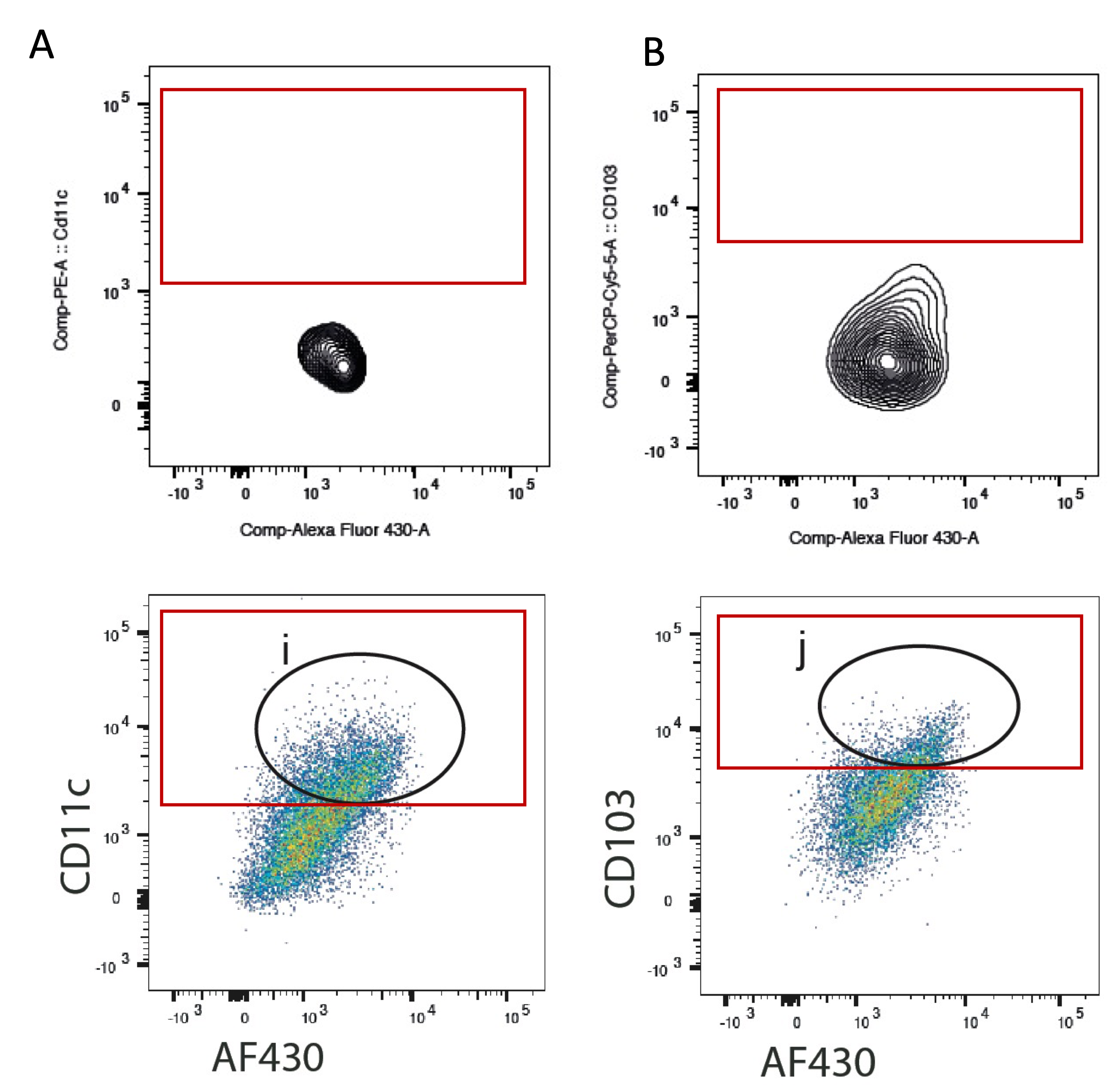


**Supplemental Figure 1: Fluorescence minus one (FMO) controls of CD11c and CD103 staining in addition to Figure 1.**

For our analyses we adjusted our gating strategy by means of FMO controls. FMO controls are the experimental cells stained with all the fluorophores minus one fluorophore. Here, we demonstrate the correctness of our gating for (A) CD11c+ and (B) CD103+ cells.


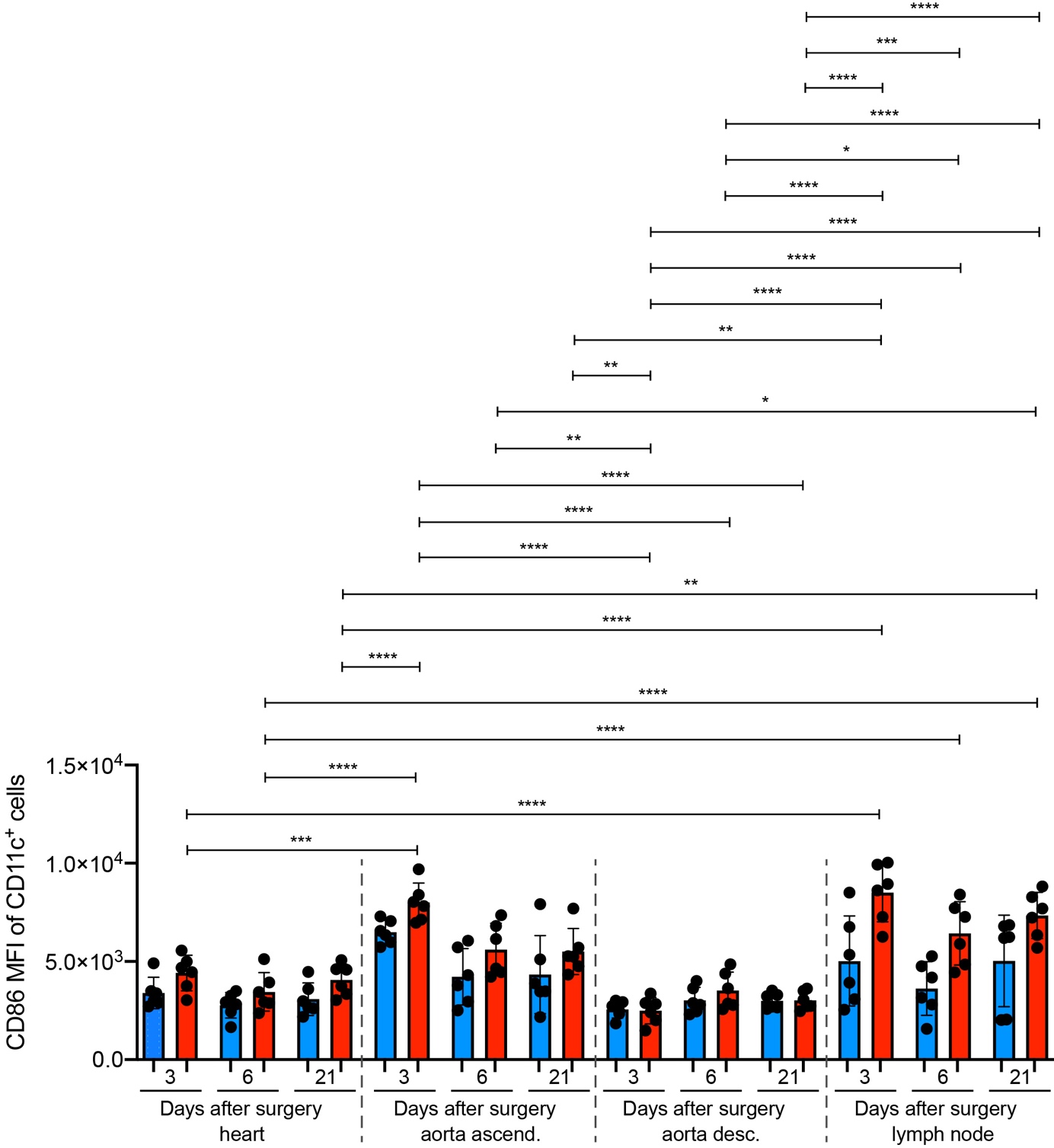


**Supplemental Figure 2: Direct comparison of CD86 MFI of CD11c^+^ cells in heart, aorta ascendens, aorta descendens and lymph node in addition to Figure 5.**

CD86 MFI was calculated from the population of CD11c^+^ classical DCs. The figure is considered in addition to figure 5 to allow a better understanding of the different CD86 MFI in the respective tissues. We compared the differences in CD86 MFI between the four different tissues at 3, 6 and 21 days after TAC or sham intervention. We depicted the statistical differences between the TAC animals in all groups. *P < 0.05, **P < 0.01, ***P < 0.001, ****P < 0.0001.
